# An Osmotic Laxative Renders Mice Susceptible to Prolonged Clostridioides difficile Colonization and Hinders Clearance

**DOI:** 10.1128/mSphere.00629-21

**Published:** 2021-09-29

**Authors:** Sarah Tomkovich, Ana Taylor, Jacob King, Joanna Colovas, Lucas Bishop, Kathryn McBride, Sonya Royzenblat, Nicholas A. Lesniak, Ingrid L. Bergin, Patrick D. Schloss

**Affiliations:** a Department of Microbiology and Immunology, University of Michigan, Ann Arbor, Michigan, USA; b Unit for Laboratory Animal Medicine, University of Michigan, Ann Arbor, Michigan, USA; Baylor College of Medicine

**Keywords:** *Clostridioides difficile*, colonization resistance, dysbiosis, microbiome

## Abstract

Antibiotics are a major risk factor for Clostridioides difficile infections (CDIs) because of their impact on the microbiota. However, nonantibiotic medications such as the ubiquitous osmotic laxative polyethylene glycol 3350 (PEG 3350) also alter the microbiota. Clinicians also hypothesize that PEG helps clear C. difficile. But whether PEG impacts CDI susceptibility and clearance is unclear. To examine how PEG impacts susceptibility, we treated C57BL/6 mice with 5-day and 1-day doses of 15% PEG in the drinking water and then challenged the mice with C. difficile 630. We used clindamycin-treated mice as a control because they consistently clear C. difficile within 10 days postchallenge. PEG treatment alone was sufficient to render mice susceptible, and 5-day PEG-treated mice remained colonized for up to 30 days postchallenge. In contrast, 1-day PEG-treated mice were transiently colonized, clearing C. difficile within 7 days postchallenge. To examine how PEG treatment impacts clearance, we administered a 1-day PEG treatment to clindamycin-treated, C. difficile-challenged mice. Administering PEG to mice after C. difficile challenge prolonged colonization up to 30 days postchallenge. When we trained a random forest model with community data from 5 days postchallenge, we were able to predict which mice would exhibit prolonged colonization (area under the receiver operating characteristic curve [AUROC] = 0.90). Examining the dynamics of these bacterial populations during the postchallenge period revealed patterns in the relative abundances of *Bacteroides*, *Enterobacteriaceae*, *Porphyromonadaceae*, *Lachnospiraceae*, and *Akkermansia* that were associated with prolonged C. difficile colonization in PEG-treated mice. Thus, the osmotic laxative PEG rendered mice susceptible to C. difficile colonization and hindered clearance.

**IMPORTANCE** Diarrheal samples from patients taking laxatives are typically rejected for Clostridioides difficile testing. However, there are similarities between the bacterial communities from people with diarrhea and those with C. difficile infections (CDIs), including lower diversity than the communities from healthy patients. This observation led us to hypothesize that diarrhea may be an indicator of C. difficile susceptibility. We explored how osmotic laxatives disrupt the microbiota’s colonization resistance to C. difficile by administering a laxative to mice either before or after C. difficile challenge. Our findings suggest that osmotic laxatives disrupt colonization resistance to C. difficile and prevent clearance among mice already colonized with C. difficile. Considering that most hospitals recommend not performing C. difficile testing on patients taking laxatives, and laxatives are prescribed prior to administering fecal microbiota transplants via colonoscopy to patients with recurrent CDIs, further studies are needed to evaluate if laxatives impact microbiota colonization resistance in humans.

## INTRODUCTION

Antibiotics are a major risk factor for Clostridioides difficile infections (CDIs) because they disrupt microbiota colonization resistance (1). However, antibiotics are not the only types of medications that disrupt the microbiota (2–4). Although other medications (proton pump inhibitors, osmotic laxatives, antimotility agents, and opioids) have been implicated as risk or protective factors for CDIs through epidemiological studies, whether the association is due to their impact on the microbiota is still unclear (5–9).

Many of the nonantibiotic medications associated with CDIs are known to modulate gastrointestinal motility, leading to either an increased or a decreased colonic transit time, which in turn also strongly impacts microbiota composition and function (10, 11). Stool consistency often serves as an approximation of intestinal motility (10). Our group has shown that when C. difficile-negative samples from patients were separated into two groups based on stool consistency, there were similar microbiota features between samples from CDI patients and those from C. difficile-negative patients with diarrhea compared to nondiarrheal samples that were C. difficile negative (12). The similar community features between CDI patients and patients with diarrhea included low alpha diversity and only 6 bacterial taxa with higher relative abundances in communities from CDI patients. These results led to the hypothesis that bacterial communities from patients experiencing diarrhea are susceptible to developing CDIs, regardless of how they developed diarrhea. For example, laxatives may disrupt colonization resistance to C. difficile.

Depending on the dose administered, osmotic laxatives can lead to diarrhea and temporarily disrupt the human intestinal microbiota (13). The ubiquitous osmotic laxative polyethylene glycol 3350 (PEG 3350) is found in Miralax, Nulytely, and Golytely and is also commonly used as bowel preparation for colonoscopies. Interestingly, previous studies have shown that treating mice with PEG alone altered the microbiota composition, reduced acetate and butyrate production, altered the mucus barrier, and rendered mice susceptible to C. difficile colonization (14–17). The mucus barrier is thought to mediate protection from CDIs by protecting intestinal epithelial cells from the toxins produced by C. difficile (18, 19). Whether laxative administration results in more severe CDIs in mice and how long mice remain colonized with C. difficile after challenge are unclear.

Beyond susceptibility, PEG is also relevant in the context of treating recurrent CDIs via fecal microbiota transplant (FMT), where a healthy microbiota is administered to the patient to restore colonization resistance. For FMTs that are delivered via colonoscopy, patients typically undergo bowel preparation by taking an osmotic laxative prior to the procedure. Many of the FMT studies to date rationalize the use of laxatives prior to the FMT (20–22) based on a 1996 case study with 2 pediatric patients where the authors suggested in the discussion that the laxative may help flush C. difficile spores and toxins from the intestine (23).

Our group has used C57BL/6 mice to characterize how antibiotics disrupt the microbiota and influence C. difficile susceptibility and clearance (24–26). Although two groups have now shown that PEG treatment alone renders mice susceptible to C. difficile (15, 17), these studies have raised additional questions regarding the dynamics and severity of infection as well as the role of laxative treatment in C. difficile clearance. Here, we characterized how long PEG-treated mice remain susceptible, whether PEG treatment results in more sustained C. difficile colonization and severe CDI than in mice treated with clindamycin, and whether PEG treatment after challenge can promote C. difficile clearance. Addressing these questions will better inform how we think about laxatives and diarrhea in the context of CDIs.

## RESULTS

### Five-day laxative treatment led to prolonged C. difficile colonization in mice.

**FIG 1:**
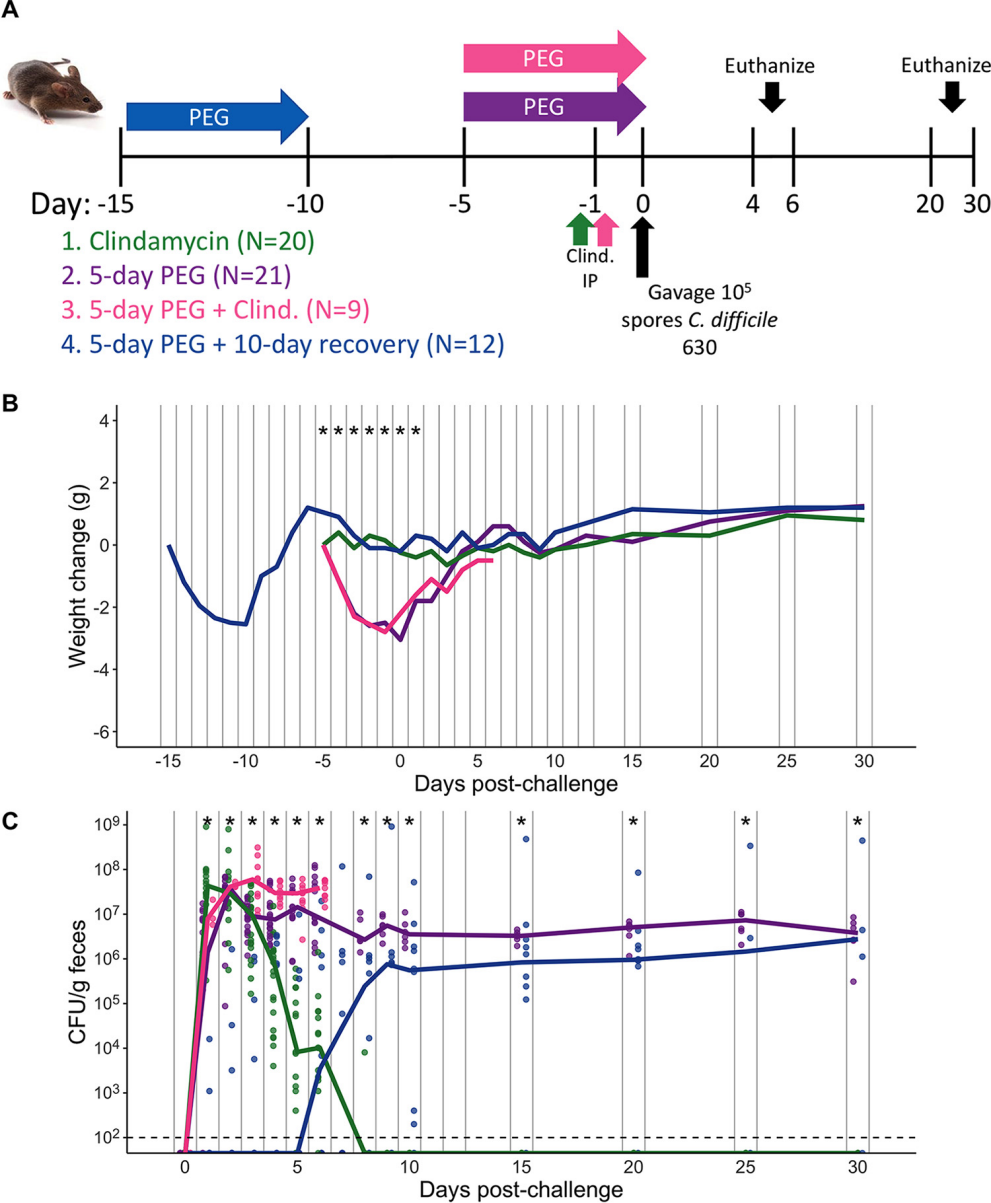
Five-day PEG treatment prolongs susceptibility, and mice become persistently colonized with C. difficile. (A) Setup of the experimental timeline for experiments with 5-day PEG-treated mice consisting of 4 treatment groups: clindamycin administered at 10 mg/kg by intraperitoneal (IP) injection (group 1), 15% PEG 3350 administered in the drinking water for 5 days (group 2), 5-day PEG plus clindamycin treatment (group 3), and 5-day PEG plus 10-day recovery treatment (group 4). All treatment groups were then challenged with 10^5^ C. difficile 630 spores. A subset of mice was euthanized at either 4 or 6 days postchallenge, and tissues were collected for histopathology analysis; the remaining mice were monitored through 20 or 30 days postchallenge. (B) Weight change from baseline weight in groups after treatment with PEG and/or clindamycin, followed by C. difficile challenge. (C) C. difficile CFU per gram of stool measured over time via serial dilutions (*n* = 10 to 59 mice per time point). The black line represents the limit of detection for the first serial dilution. CFU quantification data were not available for each mouse due to stool sampling difficulties (particularly on the day that the mice came off the PEG treatment) or early deaths. For panels B and C, lines represent the medians for each treatment group, and circles represent samples from individual mice. Asterisks indicate the specific time points on the *x* axis where the weight change or CFU per gram was significantly different (*P < *0.05) among the 3 or 4 groups indicated on the plot by the Kruskal-Wallis test with Benjamini-Hochberg correction for testing multiple time points. The data presented are from a total of 5 separate experiments.

Building off previous work that showed that treating mice with the osmotic laxative PEG 3350 rendered mice susceptible to C. difficile colonization (15, 17), we decided to test how long C. difficile colonization is sustained and how long PEG-treated mice remain susceptible to C. difficile. We compared three groups of mice treated with PEG 3350 to one group of mice treated with our standard clindamycin treatment at 10 mg/kg of body weight, which temporarily renders mice susceptible to C. difficile colonization, with mice typically clearing C. difficile within 10 days postchallenge (9, 26). All three groups of PEG-treated mice were administered a 15% PEG solution in the drinking water for 5 days. The first group received no additional treatment. The second group was also treated with clindamycin. The third group was allowed to recover for 10 days prior to challenge (Fig. 1A). The PEG treatment resulted in weight loss for the 3 groups of mice, with the greatest change in weight being observed on the fifth day of the PEG treatment. The mice recovered most of the lost weight by 5 days after treatment (Fig. 1B). After either the PEG, clindamycin, or PEG and clindamycin treatment, all mice were challenged with 10^5^ C. difficile 630 spores (Fig. 1A). All treatments rendered mice susceptible to C. difficile colonization. In contrast to the mice that received only clindamycin, PEG-treated mice remained colonized with C. difficile at a high level through 30 days postchallenge (Fig. 1C). The clindamycin-treated mice cleared C. difficile within 10 days postchallenge (Fig. 1C). It was noteworthy that PEG-treated mice were still susceptible to C. difficile colonization after a 10-day recovery period, although C. difficile was not detectable in most of the group in the initial 5 days postchallenge (Fig. 1C; see also Fig. S1A in the supplemental material). One mouse was found dead on the 6th day postchallenge, presumably due to C. difficile, as the bacterium became detectable in stool samples from that mouse on the 4th day postchallenge (Fig. S1A, mouse 10). From 8 days postchallenge onward, the density of C. difficile stabilized in the 10-day recovery group and remained high through 20 to 30 days postchallenge (Fig. 1C). Thus, osmotic laxative treatment alone was sufficient to render mice susceptible to prolonged C. difficile colonization, and PEG-treated mice remained susceptible through 10 days posttreatment.

10.1128/mSphere.00629-21.1 FIG S1 Microbiota dynamics after challenge in the 5-day PEG treatment plus 10-day recovery mice. (A) C. difficile CFU per gram over time in the stool samples collected from 5-day PEG-treated mice that were allowed to recover for 10 days prior to challenge. The same data are presented in Fig. 1C, but the data for the other 3 treatment groups have been removed, and each line represents the CFU over time for an individual mouse. Mouse 10 was found dead at 6 days postchallenge. (B) Relative abundances of eight bacterial genera from day 0 postchallenge onward in each of the 10-day recovery mice. We analyzed samples from day 0 and day 8 postchallenge, which represented the time points where mice were challenged with C. difficile and when the median relative C. difficile CFU stabilized for the group using the paired Wilcoxon signed-rank test, but no genera were significantly different after Benjamini-Hochberg correction (see Data Set S1, sheet 5, in the supplemental material). Download FIG S1, TIF file, 2.9 MB. Copyright © 2021 Tomkovich et al. 2021 Tomkovich et al. https://creativecommons.org/licenses/by/4.0/ This content is distributed under the terms of the Creative Commons Attribution 4.0 International license.

### Five-day laxative treatment differentially disrupted the fecal microbiota compared to clindamycin treatment.

**FIG 2:**
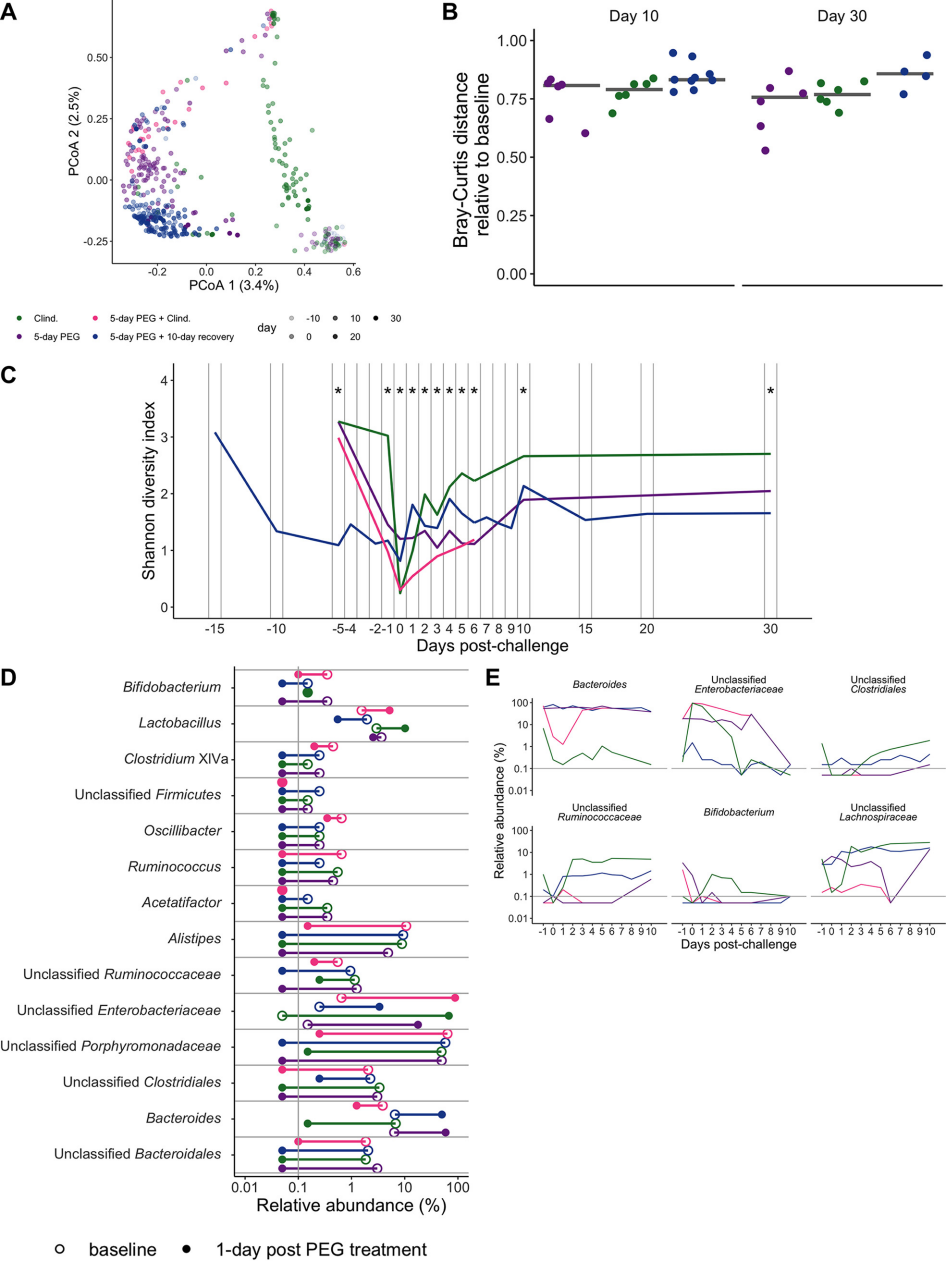
Five-day PEG treatment disrupts the stool microbiota for a long time compared to clindamycin-treated mice. (A) Principal-coordinate analysis (PCoA) of Bray-Curtis distances from stool samples collected throughout the experiment. Each circle represents a sample from an individual mouse, and the transparency of the symbol corresponds to the day postchallenge. See Data Set S1, sheet 1, in the supplemental material for PERMANOVA results. (B) Bray-Curtis distances of stool samples collected on either day 10 or day 30 postchallenge relative to the baseline sample collected for each mouse (before any drug treatments were administered). The symbols represent samples from individual mice, and the lines indicate the median value for each treatment group. (C) Shannon diversity in stool communities over time. The lines indicate the median value for each treatment group (Data Set S1, sheet 2). (D) Fourteen of the 33 genera affected by PEG treatment (Data Set S1, sheet 3). The symbols represent the median relative abundance for a treatment group at either baseline or 1 day posttreatment. Relative abundance data from paired baseline and 1-day-posttreatment stool samples from the 5-day PEG and 5-day PEG plus 10-day recovery groups were analyzed by a paired Wilcoxon signed-rank test with Benjamini-Hochberg correction for testing all identified genera. The clindamycin and 5-day PEG plus clindamycin treatment groups are shown on the plot for comparison. (E) Six of the 24 genera that were significantly different between the treatment groups over multiple time points (see Data Set S1, sheet 4, for a complete list). The 5-day PEG plus clindamycin treatment group was monitored only through 6 days postchallenge. Differences between treatment groups were identified by a Kruskal-Wallis test with Benjamini-Hochberg correction for testing all identified genera (*, *P < *0.05). The gray vertical line (D) and horizontal lines (E) indicate the limit of detection.

Since osmotic laxatives and clindamycin have previously been shown to disrupt the murine microbiota (14–17), we hypothesized that the different C. difficile colonization dynamics between mice treated with the osmotic laxative and those treated with clindamycin were due to the two drugs having differential effects on the microbiota. We profiled the stool microbiota over time by sequencing the V4 region of the 16S rRNA gene to compare changes across treatment groups. We found that time (*R*^2^ = 0.29) and treatment group (*R*^2^ = 0.21) explained half of the observed variation between fecal communities, with most of the remaining variation being explained by interactions between treatment group and other experimental variables, including time, cage, and sequencing preparation plate (permutational multivariate analysis of variance [PERMANOVA] combined *R*^2^ = 0.95; *P < *0.001) (Fig. 2A; Data Set S1, sheet 1). None of the treatment groups recovered their baseline community structure at either 10 or 30 days postchallenge, suggesting that other community features besides recovery to baseline were responsible for the prolonged C. difficile colonization in PEG-treated mice (Fig. 2B).

10.1128/mSphere.00629-21.4 DATA SET S1 Excel workbook with 19 sheets. (Sheet 1) PERMANOVA results for the stool communities from mice in the 5-day PEG subset. (Sheet 2) Shannon diversity analysis for the stool communities from mice in the 5-day PEG subset. (Sheet 3) Genera with relative abundances impacted by PEG treatment based on stool communities of 5-day PEG-treated mice. (Sheet 4) Genera with relative abundances that vary between treatment groups in the stool communities from mice in the 5-day PEG subset. (Sheet 5) Statistical analysis results for genera with relative abundances that varied in stool communities in the 5-day PEG plus 10-day recovery mice between the day 1 and day 8 time points. (Sheet 6) Shannon diversity analysis of the cecum communities from mice in the 5-day PEG experiments. (Sheet 7) PERMANOVA results for the tissue communities from mice in the 5-day PEG subset. (Sheet 8) Genera with relative abundances that vary between treatment groups in the cecum communities from mice in the 5-day PEG subset. (Sheet 9) Genera with relative abundances that vary between treatment groups in the proximal colon communities from mice in the 5-day PEG subset. (Sheet 10) Genera with relative abundances that vary between treatment groups in the distal colon communities from mice in the 5-day PEG subset. (Sheet 11) PERMANOVA results for the stool communities from mice in the 1-day PEG subset. (Sheet 12) Shannon diversity analysis of the stool communities from mice in the 1-day PEG experiments. (Sheet 13) Genera with different relative abundances between the baseline and day 1 time points in the 1-day PEG subset. (Sheet 14) Genera with different relative abundances between the baseline and day 7 time points in the 1-day PEG subset. (Sheet 15) Shannon diversity analysis of the stool communities from mice in the postchallenge PEG experiments. (Sheet 16) Richness analysis of the stool communities from mice in the postchallenge PEG experiments. (Sheet 17) PERMANOVA results for the stool communities from mice in the postchallenge PEG subset. (Sheet 18) Genera with relative abundances that vary between treatment groups in the stool communities from mice in the postchallenge PEG subset. (Sheet 19) AUROC results for the 100 different seeds from each of the 3 models tested. Download Data Set S1, XLSX file, 0.2 MB. Copyright © 2021 Tomkovich et al. 2021 Tomkovich et al. https://creativecommons.org/licenses/by/4.0/ This content is distributed under the terms of the Creative Commons Attribution 4.0 International license.

Because time and treatment group influenced most of the variation between communities, we next explored whether there were differences in community diversity and composition between treatment groups. We examined the alpha diversity dynamics by calculating the communities’ Shannon diversity. Although both clindamycin and PEG treatments decreased diversity, the Shannon diversity was lower in the groups of mice that received PEG treatment than in those that received clindamycin alone through 30 days postchallenge (Fig. 2C; Data Set S1, sheet 2). We next identified the bacterial genera whose relative abundances shifted after PEG treatment by comparing the baseline samples of mice treated with only PEG to samples from the same mice 1 day after PEG treatment. We found 18 genera whose relative abundances were altered by PEG treatment (Data Set S1, sheet 3). The majority of the bacterial relative abundances decreased after the PEG treatment, but the relative abundances among members of the *Enterobacteriaceae* and *Bacteroides* increased. The increase in the *Bacteroides* relative abundance was unique to PEG-treated mice, as the *Bacteroides* relative abundance actually decreased in clindamycin-treated mice (Fig. 2D). Finally, we identified the genera whose relative abundances differed across treatment groups over multiple time points. Of the 33 genera that were different between treatment groups, 24 genera were different over multiple time points (Fig. 2E; Data Set S1, sheet 4). Thus, PEG had a significant impact on the fecal microbiota that was maintained over time and was distinct from clindamycin treatment.

Because C. difficile was not immediately detectable in the stool samples of the PEG-treated mice that were allowed to recover for 10 days prior to challenge, we decided to examine if there were genera that changed during the postchallenge period. We compared the communities from when C. difficile shifted from undetectable at 1 day postchallenge to detectable in the stool samples, with the density stabilizing at around 8 days postchallenge (Fig. S1A). We found no genera with relative abundances that were significantly different over the two time points (Data Set S1, sheet 5). However, there were also wide variations between individual mice regarding when C. difficile became detectable (Fig. S1A) as well as the relative abundances of bacterial genera in the communities (Fig. S1B). For example, two mice had a high relative abundance of *Enterobacteriaceae* throughout the postchallenge period. One mouse died on the sixth day postchallenge, and in the other, C. difficile was present at a high density from the 4th day postchallenge onward (Fig. S1B). While we did not identify a clear signal to explain the delayed appearance of C. difficile in the 5-day PEG-treated mice that were allowed to recover for 10 days prior to challenge, the delay was striking and could reflect changes in microbial activity or metabolites that were not examined in this study.

### Five-day laxative treatment did not promote more severe CDIs despite altering the mucosal microbiota.

**FIG 3:**
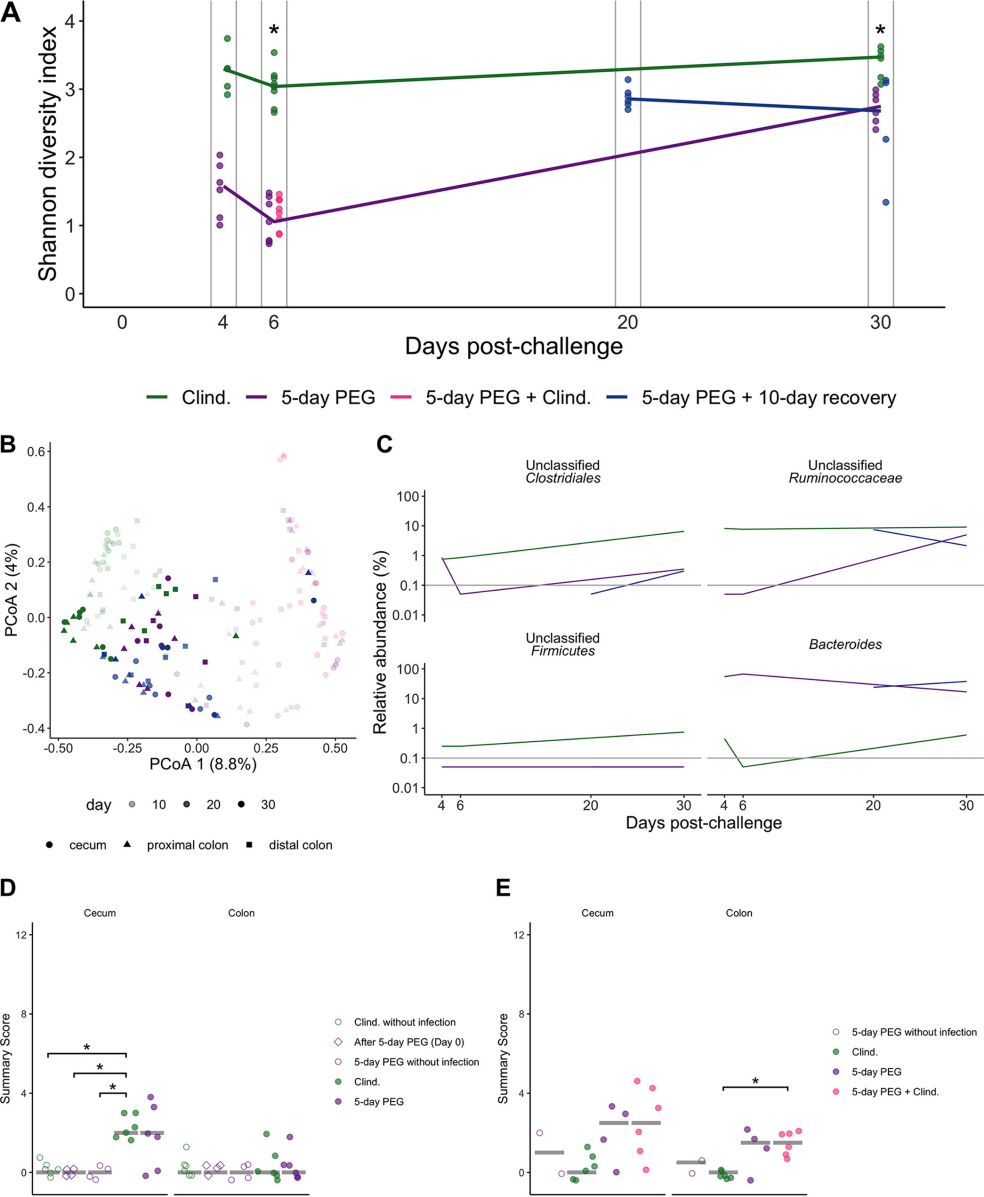
Five-day PEG treatment does not result in more severe CDIs, although the mucosal microbiota is altered. (A) Shannon diversity in cecum communities over time. The colors of the symbols and lines represent individual and median relative abundance values for four treatment groups (see Data Set S1, sheet 6, in the supplemental material). (B) PCoA of Bray-Curtis distances from mucosal samples collected throughout the experiment. The transparency of the symbols corresponds to the day postchallenge when the sample was collected. See Data Set S1, sheet 7, for PERMANOVA results. (C) Median relative abundances of the 4 genera that were significantly different between the cecum communities of different treatment groups on day 6 and day 30 postchallenge (Data Set S1, sheet 8). The gray vertical lines indicate the limit of detection. (D and E) Histopathology summary scores from cecum and colon H&E-stained tissue sections. The summary score is the total score based on evaluation of edema, cellular infiltration, and inflammation in either the cecum or colon tissue. Each category is given a score ranging from 0 to 4; thus, the maximum possible summary score is 12. The tissue for histology was collected at either 4 days (D) or 6 days (E) postchallenge, with the exception that tissues from one set of 5-day PEG-treated mock-challenged mice were collected on day 0 postchallenge. Histology data were analyzed with the Kruskal-Wallis test followed by pairwise Wilcoxon comparisons with Benjamini-Hochberg correction. *, *P < *0.05.

Given the findings from a previous study that demonstrated that PEG treatment disrupts the mucus layer and alters the immune response in mice (16), we decided to examine the impact of PEG treatment on the mucosal microbiota and CDI severity. To evaluate the mucosal microbiota, we sequenced communities associated with tissues collected from the cecum, proximal colon, and distal colon. Similar to what was observed with the stool samples, the alpha diversity was lower in the PEG-treated mice than in the clindamycin-treated mice (Fig. 3A; Data Set S1, sheet 6). The alpha diversity of the tissue-associated community increased in PEG-treated mice at 20 and 30 days postchallenge (Fig. 3A). Group (*R*^2^ = 0.33), time point (*R*^2^ = 0.11), and their interactions with other variables (cage, experiment number, and sample type) explained the majority of the variation observed in mucosal communities (PERMANOVA combined *R*^2^ = 0.83; *P < *0.05) (Fig. 3B; Data Set S1, sheet 7). We saw the greatest difference in the relative abundances of the mucosal microbiota between treatment groups (clindamycin, 5-day PEG, and 5-day PEG plus clindamycin) at 6 days postchallenge, with 10 genera that were significantly different (*P < *0.05) in all three of the tissue types that we collected (cecum, proximal colon, and distal colon) (Fig. S2A and Data Set S1, sheets 8 to 10). Interestingly, *Peptostreptococcaceae* (the family with a sequence that matches C. difficile) was one of the taxa that had a significant difference in relative abundance between treatment groups at 6 days postchallenge. This population was primarily present only in the 5-day PEG treatment group of mice and decreased in the proximal and distal colon tissues over time (Fig. S2B). By 30 days postchallenge, only the relative abundances of *Bacteroides*, *Clostridiales*, *Firmicutes*, and *Ruminococcaceae* were different between treatment groups and only in the cecum tissues (Fig. 2E and Fig. 3C; Data Set S1, sheet 8). Thus, PEG treatment had a significant impact on the mucosal microbiota, and we detected C. difficile sequences in the cecum, proximal colon, and distal colon tissue communities.

10.1128/mSphere.00629-21.2 FIG S2 PEG treatment still has a large impact on the mucosal microbiota at 6 days postchallenge. (A) Relative abundances of the 10 bacterial genera that were significantly different between treatment groups at 6 days postinfection in the cecum tissue (the relative abundances of the 10 genera were also significantly different in the proximal and distal colon tissues [see Data Set S1, sheets 8 to 10, in the supplemental material]). Each symbol represents a tissue sample from an individual mouse, and the black horizontal lines represent the median relative abundances for each treatment group. (B) Relative abundances of *Peptostreptococcaceae* in the three types of tissue sample communities over time. For panels A and B, the gray horizontal lines represent the limit of detection. Download FIG S2, TIF file, 1.8 MB. Copyright © 2021 Tomkovich et al. 2021 Tomkovich et al. https://creativecommons.org/licenses/by/4.0/ This content is distributed under the terms of the Creative Commons Attribution 4.0 International license.

Because there were differences in the mucosal microbiota, including detectable C. difficile sequences in tissues from PEG-treated mice relative to mice treated with clindamycin, we next examined the severity of C. difficile challenge by evaluating cecum and colon histopathology (27). However, we found that there was no difference in cecum and colon scores between clindamycin- and PEG-treated mice that were challenged with C. difficile at 4 days postchallenge (Fig. 3D), the time point typically examined in C. difficile 630-challenged mice (28). We also looked at 6 days postchallenge because that was when there was a large difference in C. difficile density between PEG- and clindamycin-treated mice (Fig. 1C). Although there was a slight difference in the histopathology scores of the colon between PEG- and clindamycin-treated mice, there was not a significant difference in the cecum, and the overall score was relatively low (1.5 to 2.5 out of 12) (Fig. 3E). Therefore, although PEG treatment had a disruptive effect on the mucosal microbiota, the impacts of C. difficile challenge on the cecum and colon were similar between PEG- and clindamycin-treated mice.

### C. difficile challenge did not have a synergistic disruptive effect on the microbiota of PEG-treated mice.

Because C. difficile itself can have an impact on the microbiota (29), we also sequenced the tissue and stool samples of mock-challenged mice treated with clindamycin or PEG. Examining the stool samples of the mock-challenged mice revealed similar bacterial disruptions as those of the C. difficile-challenged mice (Fig. S3A to C). Similarly, there was no difference between the communities found in the tissues of mock- and C. difficile-challenged mice (Fig. S3D to F). Thus, most of the microbiota alterations that we observed in the PEG-treated mice were a result of the laxative and not an interaction between the laxative and C. difficile.

10.1128/mSphere.00629-21.3 FIG S3 C. difficile challenge does not enhance the disruptive effect of PEG on the microbiota. (A and D) PCoAs of the Bray-Curtis distances from the stool (A) and tissue (D) communities from mock- and C. difficile-challenged treatment groups. Each symbol represents a sample from an individual mouse, with open and closed circles representing mock- and C. difficile-challenged mice, respectively. (B and E) Median Shannon diversity in stool (B) and tissue (E) communities collected over time. (C and F) Median relative abundances of genera that were significantly different between the C. difficile-challenged treatment groups in the stool (Fig. 2E) (C) and cecum tissue (Fig. 3C) (F) communities from mock- and C. difficile-challenged mice. For panels B to F, the dashed and solid lines represent the median values for mock- and C. difficile-challenged mice, respectively. For panels E and F, tissues from mock-challenged clindamycin-treated mice were collected only at 4 days postchallenge, so there is no dashed line for this group. Download FIG S3, TIF file, 2.2 MB. Copyright © 2021 Tomkovich et al. 2021 Tomkovich et al. https://creativecommons.org/licenses/by/4.0/ This content is distributed under the terms of the Creative Commons Attribution 4.0 International license.

### One-day laxative treatment resulted in transient C. difficile colonization and minor microbiota disruption.

**FIG 4:**
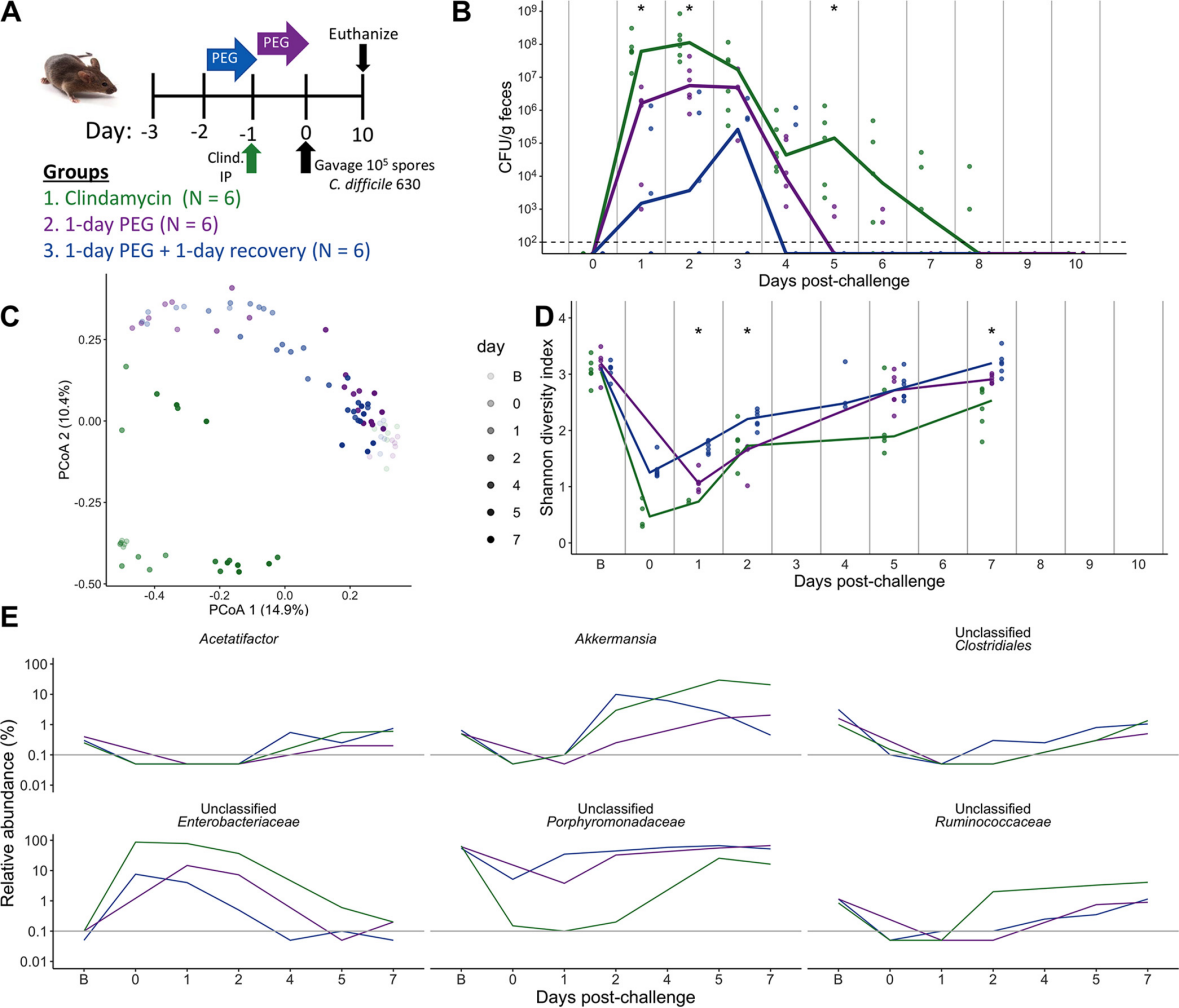
One-day PEG treatment renders mice susceptible to transient C. difficile colonization. (A) Setup of the experimental timeline for the 1-day PEG-treated mice consisting of 3 treatment groups: clindamycin administered at 10 mg/kg by intraperitoneal injection (group 1), 15% PEG 3350 administered in the drinking water for 1 day (group 2), and 1-day PEG plus 1-day recovery treatment (group 3). The three treatment groups were then challenged with 10^5^ C. difficile 630 spores. (B) C. difficile CFU per gram of stool measured over time (*n* = 12 to 18 mice per time point) by serial dilutions. The black dashed horizontal line represents the limit of detection for the first serial dilution. For panels B and D, asterisks indicate time points where there was a significant difference (*P < *0.05) between treatment groups by a Kruskal-Wallis test with Benjamini-Hochberg correction for testing multiple time points. For panels B to D, each symbol represents a sample from an individual mouse, and lines indicate the median value for each treatment group. (C) PCoA of Bray-Curtis distances from stool communities collected over time (day, *R*^2^ = 0.43; group, *R*^2^ = 0.19) (see Data Set S1, sheet 11, in the supplemental material). Symbol transparency represents the day postchallenge of the experiment. For panels C to E, the B on the day legend or day postchallenge *x* axis stands for baseline and represents the sample that was collected prior to any drug treatments. (D) Shannon diversity in stool communities over time (Data Set S1, sheet 12). (E) Median relative abundances per treatment group for 6 out of the 14 genera that were affected by treatment but recovered close to baseline levels by 7 days postchallenge (Fig. 3E; Data Set S1, sheets 13 and 14). Paired stool sample relative abundance values either at baseline and day 1 or at baseline and day 7 were analyzed by a paired Wilcoxon signed-rank test with Benjamini-Hochberg correction for testing all identified genera. Only genera that were different between baseline and 1 day postchallenge, but not between baseline and 7 days postchallenge, are shown. The gray horizontal lines represent the limit of detection.

Next, we examined how a shorter osmotic laxative perturbation would impact the microbiome and susceptibility to C. difficile. We administered either a 1-day PEG treatment, a 1-day PEG treatment with a 1-day recovery period, or clindamycin to mice before challenging them with C. difficile (Fig. 4A). In contrast to the 5-day PEG-treated mice, the 1-day PEG groups were only transiently colonized and cleared C. difficile by 7 days postchallenge (Fig. 4B). The stool communities of the 1-day PEG treatment groups were also only transiently disrupted, with Shannon diversity recovering by 7 days postchallenge (Fig. 4C and D; Data Set S1, sheets 11 and 12). We found that the relative abundances of 14 genera were impacted by treatment but recovered close to baseline levels by 7 days postchallenge, including *Enterobacteriaceae*, *Clostridiales*, *Porphyromonadaceae*, and *Ruminococcaceae* (Fig. 4E; Data Set S1, sheets 13 and 14). These findings suggest that the duration of the PEG treatment was relevant, with shorter treatments resulting in a transient loss of C. difficile colonization resistance.

### Postchallenge laxative treatment disrupted clearance in clindamycin-treated mice regardless of whether an FMT was also administered.

**FIG 5:**
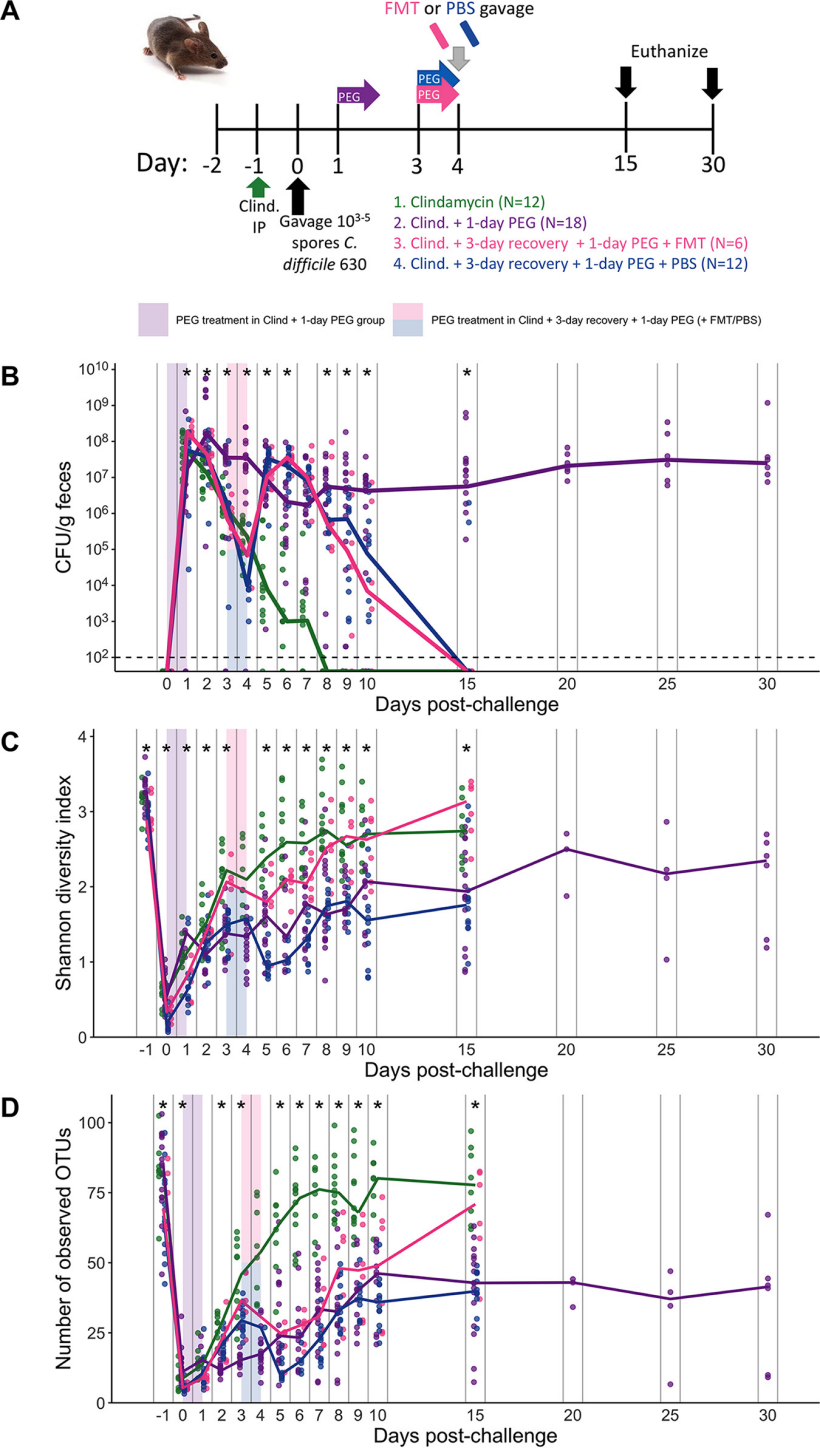
One-day PEG treatment after C. difficile challenge prolongs colonization regardless of whether an FMT is also administered. (A) Setup of the experimental timeline for experiments with postchallenge PEG-treated mice. There were 4 different treatment groups. All mice were administered 10 mg/kg clindamycin intraperitoneally (IP) 1 day before challenge with 10^3^ to 10^5^ C. difficile 630 spores. Group 1 received no additional treatment (clindamycin). For group 2, immediately after C. difficile challenge, mice received 15% PEG 3350 in the drinking water for 1 day. For groups 3 and 4, 3 days after challenge, mice received a 1-day PEG treatment and then received either 100 μl of a fecal microbiota transplant (group 3) or PBS (group 4) solution by oral gavage. Mice were monitored through 15 to 30 days postchallenge (only the post-CDI 1-day PEG group was monitored through 30 days postchallenge). (B) CFU of C. difficile per gram of stool measured over time via serial dilutions. The black line represents the limit of detection for the first serial dilution. (C and D) Shannon diversity (C) and richness (D) in stool communities over time (see Data Set S1, sheets 15 and 16, in the supplemental material). For panels B to D, each symbol represents a stool sample from an individual mouse, with the lines representing the median value for each treatment group. Asterisks indicate time points with significant differences (*P < *0.05) between groups by the Kruskal-Wallis test with Benjamini-Hochberg correction for testing multiple times points. Colored rectangles indicate the 1-day PEG treatment period for applicable groups. The data presented are from a total of 3 separate experiments.

Since a 1-day PEG treatment resulted in a milder perturbation of the microbiota, we decided to use the 1-day treatment to examine the hypothesis that PEG helps to flush C. difficile spores from the intestine. This hypothesis is proposed in the discussion sections of FMT studies where bowel prep is part of the preparation undergone by patients receiving FMTs via colonoscopy (20–23). To examine the impact of PEG treatment on C. difficile clearance, we treated 4 groups of mice with clindamycin and then challenged all mice with C. difficile before administering the following treatments: no additional treatment, 1-day PEG treatment immediately after challenge, and 1-day PEG treatment 3 days after challenge followed by the administration of either an FMT or a phosphate-buffered saline (PBS) solution by oral gavage (Fig. 5A). Contrary to the hypothesis, all groups of mice that received PEG exhibited prolonged C. difficile colonization (Fig. 5B).

**FIG 6:**
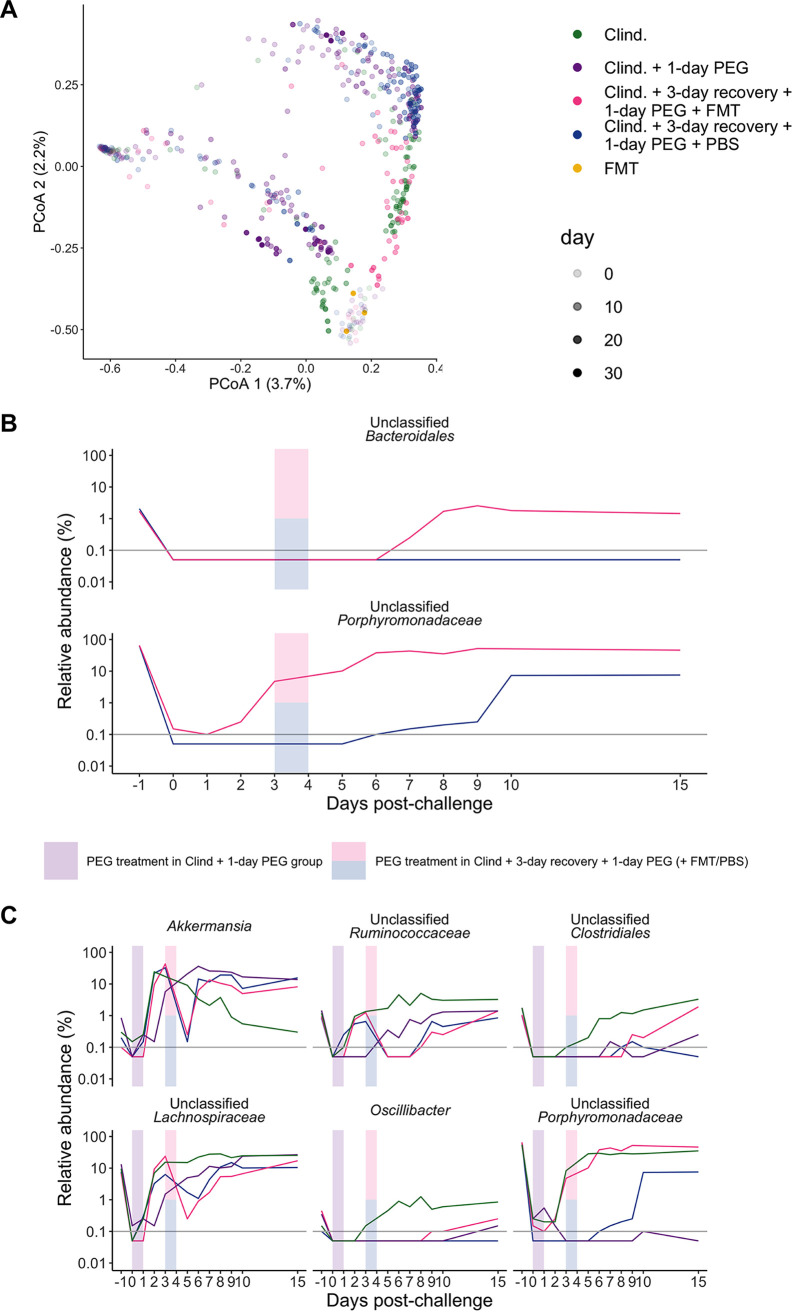
For 1-day PEG treatment after C. difficile challenge of mice that also received an FMT, only some bacterial genera were restored. (A) PCoA of Bray-Curtis distances from stool samples collected over time as well as the FMT solution that was administered to one of the treatment groups. Each circle represents an individual sample, and the transparency of the circle corresponds to the day postchallenge. See Data Set S1, sheet 17, in the supplemental material for PERMANOVA results. (B) Median relative abundances of 2 genera that were significantly different over multiple time points in mice that were administered either the FMT or PBS solution via gavage. (C) Median relative abundances of the top 6 out of 24 genera that were significant over multiple time points, plotted over time (see Data Set S1, sheet 18, for a complete list). For panels B and C, colored rectangles indicate the 1-day PEG treatment period for applicable groups. Gray horizontal lines represent the limit of detection. Differences between treatment groups were identified by a Kruskal-Wallis test with Benjamini-Hochberg correction for testing all identified genera. For pairwise comparisons of the groups (B), we performed pairwise Wilcoxon comparisons with Benjamini-Hochberg correction for testing all combinations of group pairs.

We were also interested in exploring whether PEG might help with engraftment in the context of FMTs. An FMT was prepared under anaerobic conditions using stool collected from the same group of mice before treatment, representing the baseline community. The FMT appeared to partially restore Shannon diversity but not richness (Fig. 5C and D; Data Set S1, sheets 15 and 16). Similarly, we saw some overlap between the communities of mice that received FMT and those of the mice treated with only clindamycin after 5 days postchallenge (Fig. 6A; Data Set S1, sheet 17). The increase in Shannon diversity suggests that the FMT had an impact on the microbiota despite seeing prolonged C. difficile colonization in the FMT-treated mice. However, only the relative abundances of *Bacteroidales* and *Porphyromonadaceae* consistently differed between the mice that received an FMT and those that received PBS (Fig. 6B). Overall, we found that the relative abundances of 24 genera were different between treatment groups over multiple time points (Data Set S1, sheet 18). For example, the relative abundance of *Akkermansia* was increased and the relative abundances of *Ruminococcaceae*, *Clostridiales*, *Lachnospiraceae*, and *Oscillibacter* were decreased in mice that received PEG after C. difficile challenge relative to clindamycin-treated mice (Fig. 6C). In sum, administering PEG actually prolonged C. difficile colonization, including in mice that received an FMT, which restored only 2 bacterial genera.

### Five-day-postchallenge community data can predict which mice will have prolonged C. difficile colonization.

**FIG 7:**
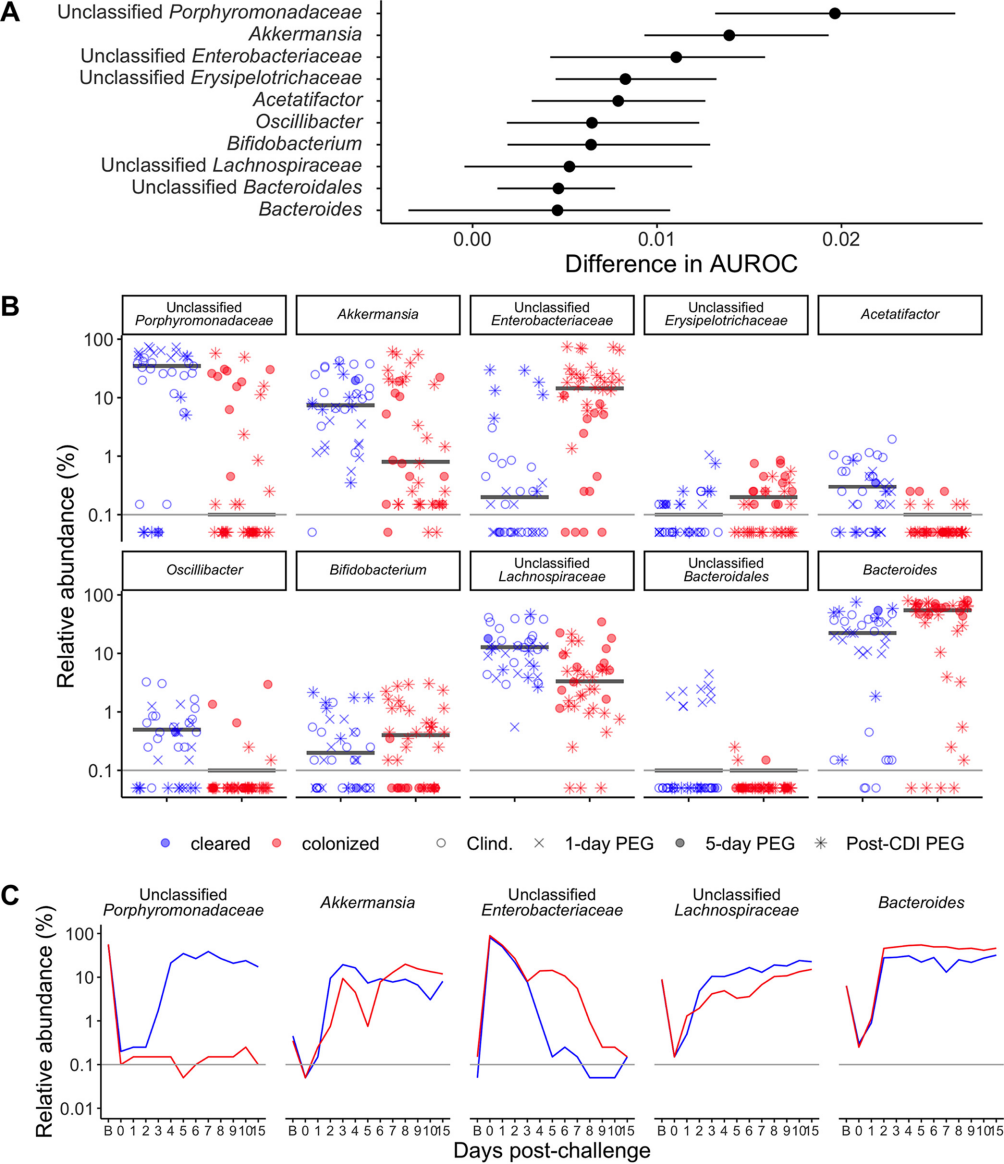
Specific microbiota features associated with prolonged C. difficile colonization in PEG-treated mice. (A) Top 10 bacteria that contributed to the random forest model trained on 5-day-postchallenge community relative abundance data, predicting whether mice would still be colonized with C. difficile at 10 days postchallenge. The medians (points) and interquartile ranges (lines) changed in the AUROC analysis when the bacteria were left out of the model by permutation feature importance analysis. (B) Median relative abundances of the top 10 bacteria that contributed to the random forest classification model at 5 days postchallenge. Red indicates the mice that were still colonized with C. difficile, while blue indicates mice that cleared C. difficile at 10 days postchallenge, and the black horizontal lines represent the median relative abundances for the two categories. Each symbol represents a stool sample from an individual mouse, and the shape of the symbol indicates whether the PEG-treated mice received a 5-day (Fig. 1 to 3), 1-day (Fig. 4), or postchallenge PEG (Fig. 5 and 6) treatment. (C) Median relative abundances of the 5 genera with a >1% median relative abundance in the stool community over time. For panels B and C, the gray horizontal lines represent the limit of detection.

After identifying bacteria associated with the 5-day, 1-day, and postchallenge 1-day PEG treatments, we examined the bacteria that influenced prolonged C. difficile colonization. We trained 3 machine learning models (random forest, logistic regression, and support vector machine) with bacterial community data from 5 days postchallenge to predict whether the mice were still colonized with C. difficile at 10 days postchallenge. We chose to predict the status based on communities at 5 days postchallenge because that was the earliest time point where we saw a treatment effect in the postchallenge 1-day PEG experiments. The random forest model had the highest performance (median area under the receiver operating characteristic curve [AUROC] = 0.90) (Data Set S1, sheet 19) and indicated that the 5-day-postchallenge microbiota was an excellent predictor of prolonged C. difficile colonization. Next, we performed a permutation importance test to identify the bacteria that were the top contributors to the random forest model for predicting prolonged C. difficile colonization. We selected 10 genera that contributed the most to our model’s performance (Fig. 7A) and examined their relative abundances at 5 days postchallenge, the time point used to predict C. difficile colonization status on day 10 (Fig. 7B). Next, we focused on the 5 genera that had a >1% relative abundance in either the cleared or colonized mice and examined how the bacteria changed over time. We found that *Enterobacteriaceae* and *Bacteroides* tended to consistently have high relative abundances, the relative abundance of *Akkermansia* was initially low and then increased, and *Porphyromonadaceae* and *Lachnospiraceae* had low relative abundances in the mice with prolonged colonization compared to the mice that cleared C. difficile (Fig. 7C). Together, these results suggest that a combination of low- and high-abundance bacterial genera influences the prolonged colonization observed in 5-day PEG- and postchallenge 1-day PEG-treated mice.

## DISCUSSION

**FIG 8:**
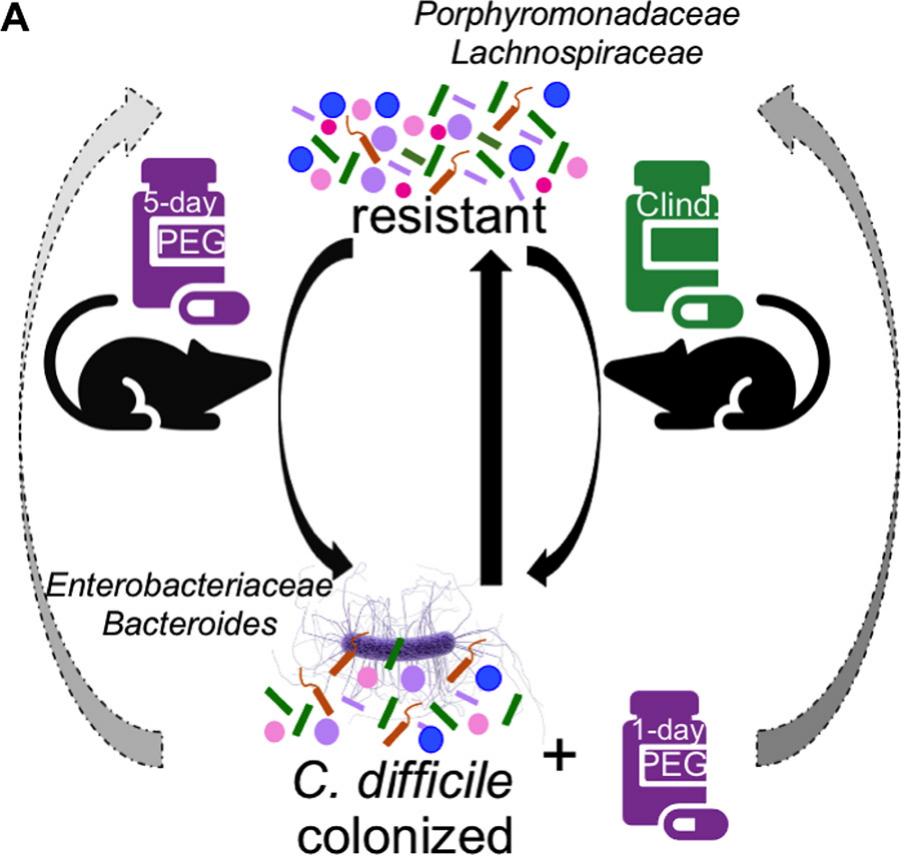
Schematic summarizing findings. The gut microbiota of our laboratory’s C57BL/6 mice is resistant to C. difficile, but treatment with either clindamycin or the osmotic laxative PEG 3350 renders the mice susceptible to C. difficile colonization. Recovery of colonization resistance in clindamycin-treated mice is relatively straightforward, and the mice clear C. difficile within 10 days postchallenge. However, recovery of colonization resistance was delayed for mice that received either a 5-day PEG pretreatment or a 1-day PEG post-C. difficile-challenge treatment. We found that increased relative abundances of *Porphyromonadaceae* and *Lachnospiraceae* were associated with C. difficile clearance, while increased relative abundances of *Enterobacteriaceae* and *Bacteroides* were associated with prolonged C. difficile colonization.

While the disruptive effect of antibiotics on C. difficile colonization resistance is well established, the extent to which other drugs such as laxatives disrupt colonization resistance was unclear. By monitoring mice treated with an osmotic laxative over time, we found that a 5-day PEG treatment before challenge resulted in prolonged C. difficile colonization, while a 1-day PEG treatment resulted in transient colonization without the use of antibiotics. The differences in C. difficile colonization dynamics between the 5- and 1-day PEG-treated mice were associated with differences in the degree to which treatments disrupted the microbiota. Additionally, the intestinal communities of 5-day PEG-treated mice did not regain colonization resistance after a 10-day recovery period. In contrast to the other 5-day PEG treatment groups, C. difficile was not immediately detectable in the stool samples of most of the mice in the 10-day recovery group. We also examined the impact of PEG treatment after C. difficile challenge. In opposition to the hypothesis suggested by the literature, we found that PEG treatment prolonged colonization relative to mice that received only clindamycin treatment. We identified patterns in the relative abundances of *Bacteroides*, *Enterobacteriaceae*, *Akkermansia*, *Porphyromonadaceae*, and *Lachnospiraceae* that were associated with prolonged C. difficile colonization (Fig. 8). Overall, our results demonstrated that osmotic laxative treatment alone rendered mice susceptible to C. difficile colonization, and the duration of colonization depended on the length of PEG treatment and whether treatment was administered before or after challenge.

In addition to altering composition, laxative treatment may alter microbiota-produced metabolites. A previous study demonstrated that a 5-day treatment of 10% PEG depleted acetate and butyrate and increased succinate compared to untreated mice (15). While we did not perform a metabolomic analysis, we saw that bacteria known to produce beneficial metabolites were depleted in mice that cleared C. difficile compared to mice with prolonged colonization (Fig. 7B). For example, Oscillibacter valericigenes can produce the short-chain fatty acid (SCFA) valerate (30), and separate studies demonstrated that valerate is depleted after clindamycin treatment and inhibits C. difficile growth *in vitro* and in C57BL/6 mice (31, 32). Similarly, *Acetatifactor* can produce acetate and butyrate (33), SCFAs that are decreased in mice with prolonged C. difficile infection after antibiotic treatment (34). Thus, protective bacteria and their metabolites could be depleted by osmotic laxative treatment depending on the timing and duration of treatment.

One possible explanation for the prolonged C. difficile colonization in 5-day PEG-treated mice is the bacterium’s persistence in the mucosal compartment either within host cells (35) or together with other bacteria. In fact, it has been hypothesized that C. difficile biofilms may serve as reservoirs for recurrent infections (36), and C. difficile biofilms in the mucus layer were recently identified in patients as aggregates with Fusobacterium nucleatum (37). There was an interesting pattern of increased *Enterobacteriaceae*, *Bacteroides*, and C. difficile in both the stool and mucosal communities of PEG-treated mice, suggesting potential synergy. *Bacteroides* has the potential to degrade mucus, and the osmotic laxative may have allowed *Bacteroides* to colonize the mucosal niche by degrading mucin glycans with glycosyl hydrolases that are absent in C. difficile (38). *Bacteroides* persistence in the mucosal tissue might also have helped *Enterobacteriaceae* to colonize the region, as synergy between mucus-degrading Bacteroides fragilis and Escherichia coli has previously been described (39). A separate study demonstrated that C. difficile was present in the outer mucus layer and associated with *Enterobacteriaceae* and *Bacteroidaceae* using fluorescent *in situ* hybridization (FISH) staining (40). However, protective roles for *Bacteroides* have also been demonstrated. For example, B. fragilis prevented CDI morbidity in a mouse model and inhibited C. difficile adherence *in vitro* (41). In coculture experiments, Bifidobacterium longum decreased C. difficile biofilm formation, while Bacteroides thetaiotaomicron enhanced biofilm formation (42), and Bacteroides dorei reduced C. difficile growth in a 9-species community *in vitro* (43). Therefore, whether *Bacteroides* is detrimental or beneficial in the context of C. difficile infection or colonization is still unclear, but the niche and interactions with other bacteria may contribute.

*Akkermansia* is also a mucin degrader with potentially beneficial or detrimental roles depending on context in other diseases (44, 45). In our study, the relative abundance of *Akkermansia* shifted over time between groups of mice that cleared C. difficile and those that had prolonged colonization. In the stool, it was initially increased in mice that cleared C. difficile but shifted after 5 days postchallenge so that it was increased in mice that had prolonged colonization. In the context of CDIs, some studies suggest a protective role (46, 47), while others suggest a detrimental role because *Akkermansia* was positively correlated with C. difficile (48–51). Because the relative abundance of *Akkermansia* was dynamic in our study, it is unclear whether *Akkermansia* helps with the clearance of C. difficile or allows it to persist. A better understanding of how C. difficile interacts with the mucosal microbiota may lead to insights into CDIs, asymptomatic C. difficile carriage, and colonization resistance.

Despite identifying an altered compositional profile that included high relative abundances of the C. difficile sequence in the mucosal tissues of mice treated with 5 days of PEG compared to the clindamycin group, we did not see a difference in histopathology scores between the groups. One reason why there was no difference could be the C. difficile strain used: C. difficile 630 results in mild histopathology summary scores in mice compared to VPI 10463 despite both strains producing toxin in mice (52). Part of our hypothesis for why there could have been increased histopathology scores in PEG-treated mice was because PEG was previously shown to disrupt the mucus layer in mice. However, recent studies demonstrated that broad-spectrum antibiotics can also disrupt the host mucosal barrier in mice (53, 54). Further research is needed to tease out the interplay between medications that influence the mucus layer and different strains of C. difficile in the context of CDIs.

The extent to which laxatives disrupt C. difficile colonization resistance in human patients is unclear based on the current literature, the main difficulty being that most hospitals recommend not performing C. difficile testing if the patient is currently taking a laxative. This recommendation is in accordance with Infectious Diseases Society of America and Society for Healthcare Epidemiology of America guidelines (55). The rationale behind the recommendation is that patients taking laxatives may be asymptomatically colonized with C. difficile, resulting in unnecessary antibiotic treatment (56–58). Furthermore, some studies identified laxatives as a risk factor for developing CDIs or recurrent CDIs (59–61), and a recent study found that the proportions of severe CDIs were similar between patients taking and those not taking laxatives (62). However, there have also been some studies that suggest that laxatives are not a risk factor for developing CDIs (63, 64). Although it is unclear whether laxatives impact CDI susceptibility in human patients, it is clear that laxatives also have a transient impact on the human microbiota (13, 65–68). Additional studies to examine the relationship between laxatives, C. difficile colonization, and CDIs are warranted.

Considering that laxatives are also used to prepare patients when administering fecal microbiota transplants via colonoscopy to treat recurrent CDIs, it will be important to determine whether osmotic laxatives impact C. difficile clearance in the human intestinal tract. It is still unclear what the best administration route is because there have been no studies designed to evaluate the best administration route for FMTs (69). Nevertheless, results from the FMT National Registry where 85% of FMTs were delivered by colonoscopy demonstrate that FMTs are highly effective treatments for recurrent CDIs, with 90% achieving resolution in the 1-month follow-up window (70). A surprising number of studies continue to hypothesize that PEG or bowel preparation can clear C. difficile spores and toxins despite the paucity of supporting evidence (20–23). There was even a clinical trial (ClinicalTrials.gov identifier NCT01630096) designed to examine whether administering PEG 3350 (Nulytely) prior to antibiotic treatment reduced disease severity that started recruitment in 2012 (71), but no results have been posted to date. Here, we sought to evaluate the impact of treating C. difficile-colonized mice with PEG (with or without FMT) and found that clearance was delayed. Further studies are needed to understand the impact of osmotic laxatives on C. difficile colonization resistance and clearance in human patients receiving FMTs.

We have demonstrated that osmotic laxative treatment alone has a substantial impact on the microbiota and rendered mice susceptible to prolonged C. difficile colonization, in contrast to clindamycin-treated mice. The duration and timing of the laxative treatment impacted the duration of C. difficile colonization, with only 5-day PEG and postchallenge 1-day PEG treatments prolonging colonization compared to clindamycin-treated mice. Further studies are warranted to ascertain whether laxatives have a similar impact on C. difficile colonization resistance of the human microbiota.

## MATERIALS AND METHODS

### Animals.

All experiments were approved by the University of Michigan Animal Care and Use Committee (protocol numbers PRO00006983 and PRO00008975). All mice were C57BL/6 and part of the Schloss laboratory colony, which was established in 2010 with mice donated from Vincent Young’s laboratory colony (established with mice purchased from The Jackson Laboratory in 2002). We used 7- to 19-week-old female mice for all experiments. This allowed us to break up littermates and distribute them as evenly as possible across treatment groups in order to minimize microbiota differences prior to starting treatments with medications. During the experiment, mice were housed at a density of 2 to 3 mice per cage, with the majority of cages being limited to 2 mice.

### Drug treatments.

For PEG treatment groups, 15% PEG 3350 (Miralax) was administered in the drinking water for either 5- or 1-day periods depending on the experiment. The PEG solution was prepared fresh every 2 days in distilled water and administered to the mice in water bottles. Clindamycin treatment groups received distilled water in water bottles during the PEG treatment periods, with the water being changed at the same frequency. For clindamycin treatment, groups of mice received 10 mg/kg clindamycin (Sigma-Aldrich) via intraperitoneal injection. All PEG treatment groups received a sham intraperitoneal injection containing filter-sterilized saline.

### C. difficile challenge model.

Mice were challenged with 25 μl containing 10^5^ C. difficile 630 spores, except for 1 experiment where the concentration was 10^3^ spores (Fig. 5A). All mock-challenged mice received 25 μl of a vehicle solution (ultrapure water). A Dymax stepper pipette was used to administer the same challenge dose to mice via oral gavage. Mice were weighed daily throughout the experiment, and stool was collected for quantifying C. difficile CFU and 16S rRNA gene sequencing. Fresh stool samples were collected from each mouse and split into two separate tubes. One tube was transferred to an anaerobic chamber on the same day that the sample was collected to quantify C. difficile, while the other tube was snap-frozen in liquid nitrogen and stored at −80°C for 16S rRNA sequencing. There were two groups of mice that received either a PBS or fecal microbiota transplant (FMT) gavage after PEG treatment. The fecal microbiota transplant was prepared with stool samples collected from the mice in the experiment prior to the start of any treatments. The stool samples were transferred to an anaerobic chamber and diluted 1:10 in reduced PBS, and glycerol was added to make a 15% glycerol solution. The solution was then aliquoted into tubes and stored at −80°C until the day of the gavage. An aliquot of both the FMT and PBS solutions was also set aside at −80°C for 16S rRNA gene sequencing. On the day of the gavage, aliquots were thawed and centrifuged at 7,500 rpm for 1 min. The supernatant was then transferred to a separate tube to prevent the gavage needle from clogging with debris during the gavage. The PBS solution that was administered to the other group was also 15% glycerol. Each mouse was administered 100 μl of either the FMT or PBS solution via gavage. When we refer to mice that cleared C. difficile, we mean that no C. difficile was detected in the first serial dilution (limit of detection of 100 CFU). In some experiments, we collected tissues for 16S rRNA gene sequencing, histopathology, or both. For 16S rRNA gene sequencing, we collected small snips of cecum, proximal colon, and distal colon tissues in microcentrifuge tubes; snap-froze the samples in liquid nitrogen; and stored them at −80°C. For histopathology, cecum and colon tissues were placed into separate cassettes, fixed, and then submitted to McClinchey Histology Labs (Stockbridge, MI) for processing, embedding, and hematoxylin and eosin (H&E) staining.

### C. difficile quantification.

Stool samples from mice were transferred to an anaerobic chamber and serially diluted in reduced PBS. Serial dilutions were plated onto taurocholate-cycloserine-cefoxitin-fructose agar (TCCFA) plates and counted after 24 h of incubation at 37°C. Stool samples collected from the mice on day 0 postchallenge were also plated onto TCCFA plates to ensure that mice were not already colonized with C. difficile prior to challenge.

### 16S rRNA gene sequencing.

Stool samples were stored at −80°C and placed into 96-well plates for DNA extractions and library preparation. DNA was extracted using the DNeasy PowerSoil HTP 96 kit (Qiagen) and an EpMotion 5075 automated pipetting system (Eppendorf). For library preparation, each plate had a mock community control (ZymoBIOMICS microbial community DNA standards) and a negative control (water). The V4 region of the 16S rRNA gene was amplified with AccuPrime *Pfx* DNA polymerase (Thermo Fisher Scientific) using custom barcoded primers, as previously described (72). The PCR amplicons were normalized (SequalPrep normalization plate kit; Thermo Fisher Scientific), pooled and quantified (Kapa library quantification kit; Kapa Biosystems), and sequenced with the MiSeq system (Illumina).

### 16S rRNA gene sequence analysis.

All sequences were processed with mothur (v.1.43) using previously published protocols (72, 73). Paired sequencing reads were combined and aligned with the SILVA (v.132) reference database (74), and taxonomy was assigned with a modified version of the Ribosomal Database Project reference sequences (v.16) (75). The error rate for our sequencing data was 0.0559% based on the 17 mock communities that we ran with the samples. Samples were rarefied to 1,000 sequences, 1,000 times for alpha and beta diversity analyses, in order to account for uneven sequencing across samples. All but 3 of the 17 water controls had fewer than 1,000 sequences. Principal-coordinate analyses (PCoAs) were performed based on Bray-Curtis index distance matrices. Permutational multivariate analyses of variance (PERMANOVAs) were performed on mothur-generated Bray-Curtis distance matrices with the adonis function from the vegan R package (76).

### Histopathology.

H&E-stained sections of cecum and colon tissues collected at either 0, 4, or 6 days postchallenge were coded to be scored in a blind manner by a board-certified veterinary pathologist (I. L. Bergin). Slides were evaluated using a scoring system developed for mouse models of C. difficile infection (52). Each slide was evaluated for edema, cellular infiltration, and inflammation and given a score ranging from 0 to 4. The summary score was calculated by combining the scores from the 3 categories and ranged from 0 to 12.

### Classification model training and evaluation.

We used the mikropml package to train and evaluate models to predict C. difficile colonization status at 10 days postchallenge where mice were categorized as either cleared or colonized (77, 78). We removed the C. difficile genus relative abundance data prior to training the model. Input community relative abundance data at the genus level from 5 days postchallenge were used to generate random forest, logistic regression, and support vector machine classification models to predict C. difficile colonization status at 10 days postchallenge. To accommodate the small number of samples in our data set, we used 50% training and 50% testing splits with repeated 2-fold cross-validation of the training data for hyperparameter tuning. Permutation importance was performed as described previously (79) using mikropml (77, 78) with the random forest model because it had the highest AUROC value.

### Statistical analysis.

R (v.4.0.2) and the tidyverse package (v.1.3.0) were used for statistical analysis (80, 81). Kruskal-Wallis tests with Benjamini-Hochberg correction for testing multiple time points were used to analyze differences in C. difficile CFU, mouse weight changes, and alpha diversity between treatment groups. Paired Wilcoxon signed-rank tests were used to identify genera impacted by treatments on matched pairs of samples from 2 time points. Bacterial relative abundances that varied between treatment groups at the genus level were identified with the Kruskal-Wallis test with Benjamini-Hochberg correction for testing all identified operational taxonomic units (OTUs), followed by pairwise Wilcoxon comparisons with Benjamini-Hochberg correction.

### Data availability.

The 16S rRNA sequencing data have been deposited in the National Center for Biotechnology Information Sequence Read Archive (BioProject accession no. PRJNA727293). Code for data analysis and generating this paper with the accompanying figures is available at https://github.com/SchlossLab/Tomkovich_PEG3350_mSphere_2021.
