# Supplementary figures and images for: An Osmotic Laxative Renders Mice Susceptible to Prolonged Clostridioides difficile Colonization and Hinders Clearance
Source: mSphere. 2021 Sep 29;6(5):e00629-21. doi: 10.1128/mSphere.00629-21 (PMC8550136; doi:10.1128/mSphere.00629-21)

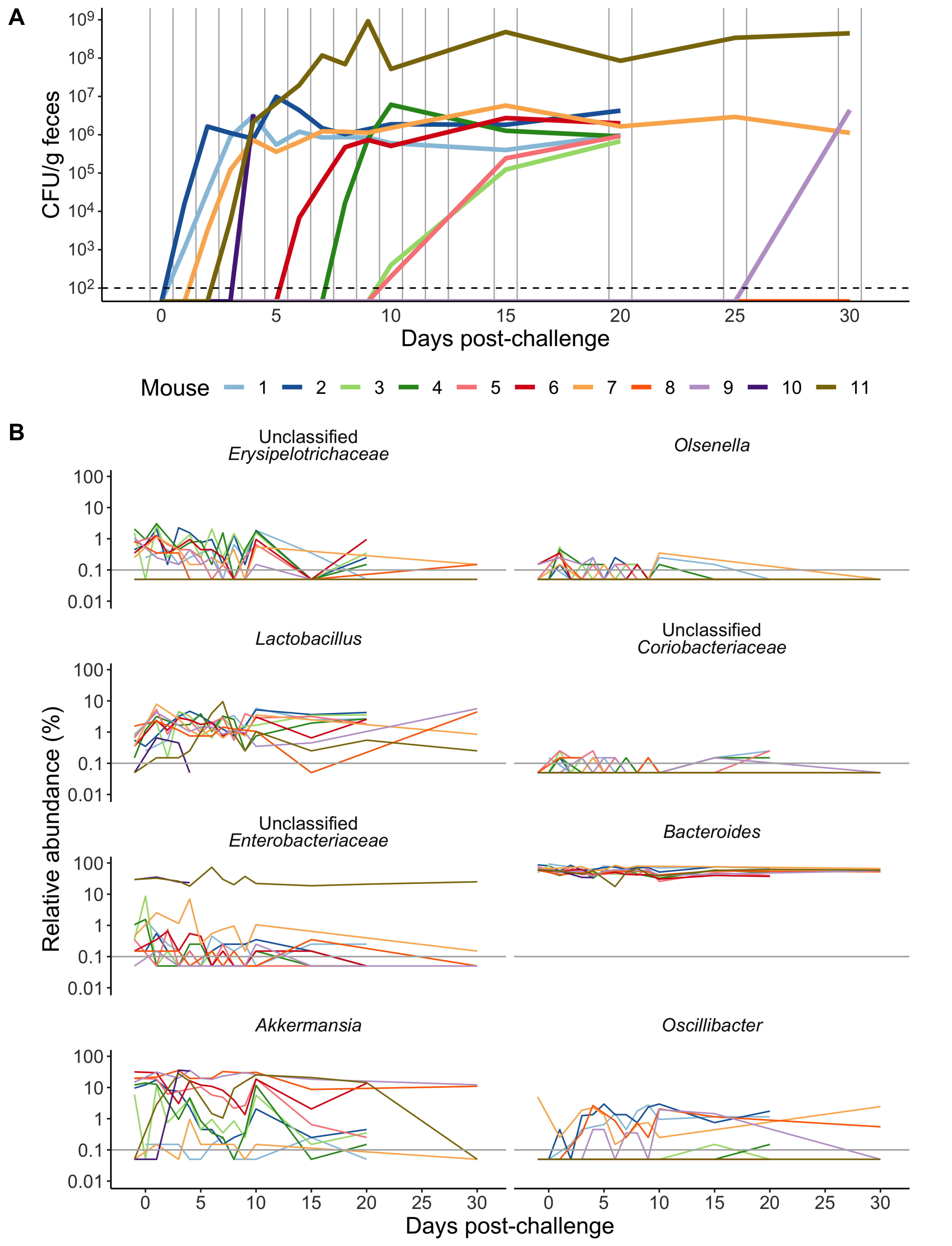

Supplement: FIG S1 [file msphere.00629-21-sf001.tif]

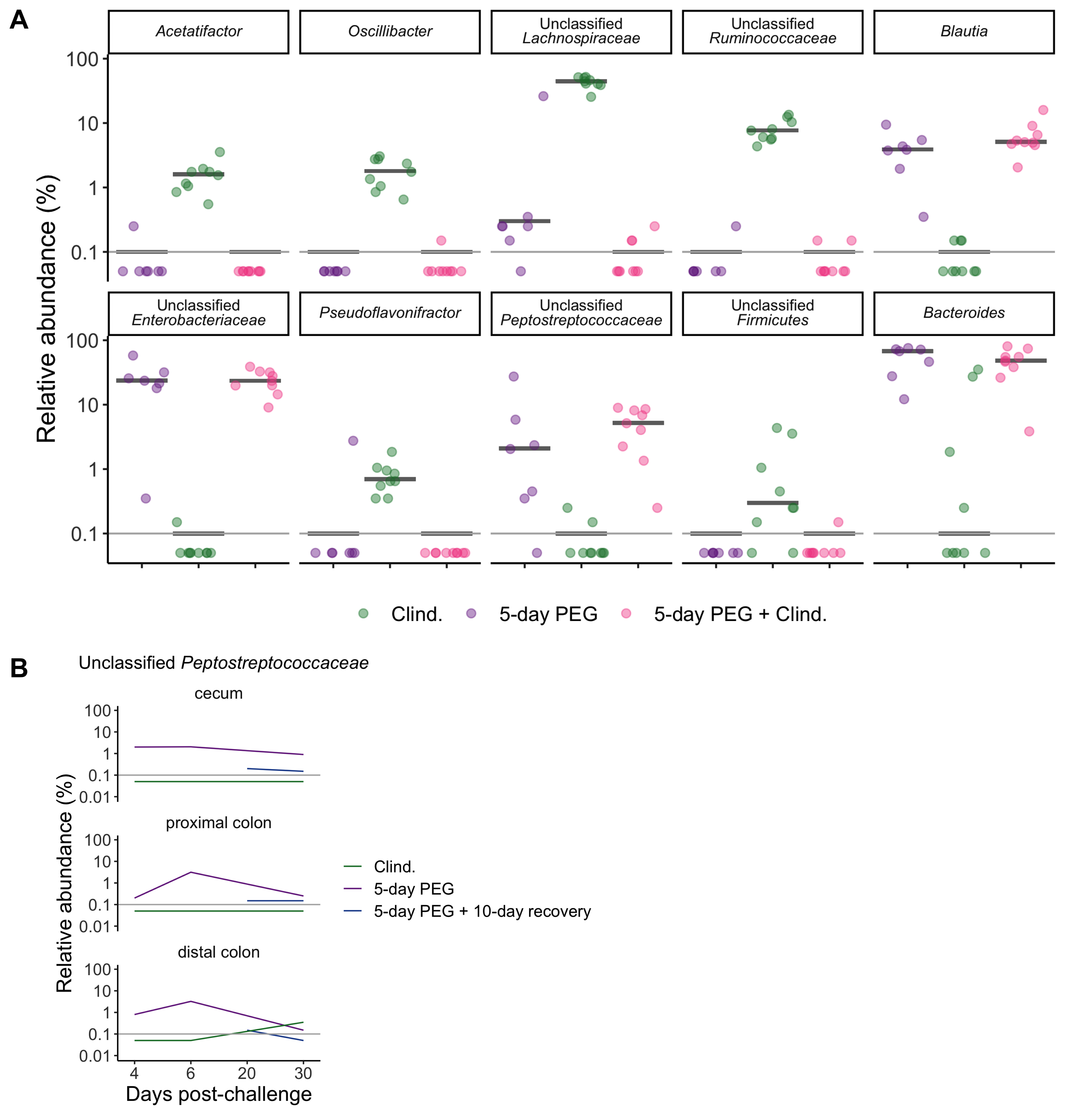

Supplement: FIG S2 [file msphere.00629-21-sf002.tif]

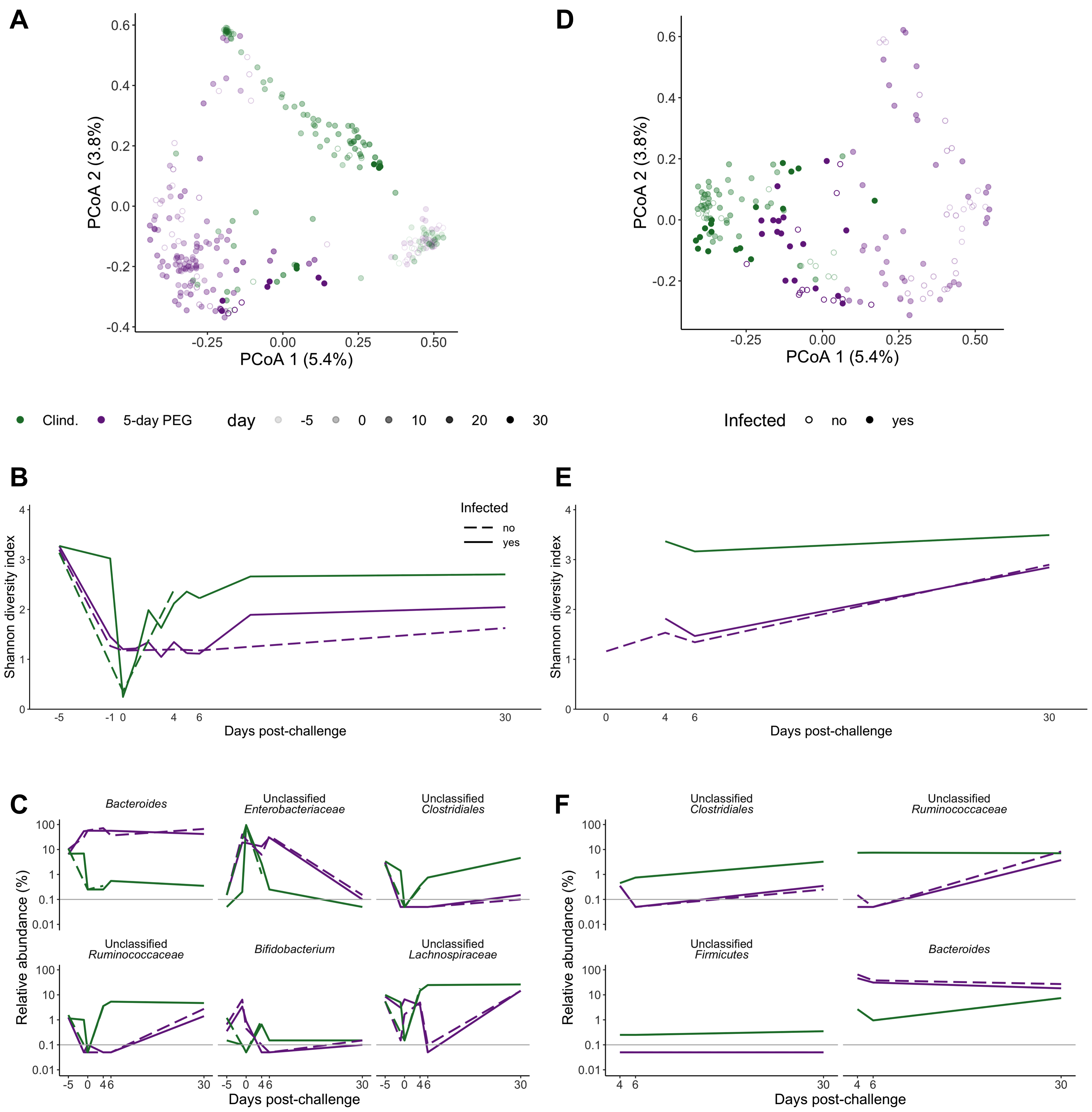

Supplement: FIG S3 [file msphere.00629-21-sf003.tif]
